# Novel laparoscopic methods for inguinal hernia post pelvic fracture: A case report

**DOI:** 10.1016/j.ijscr.2020.01.048

**Published:** 2020-02-06

**Authors:** Yusuke Tsunetoshi, Akihiro Usui, Koichi Inukai, Motohiko Yamada, Masanori Kawamoto, Hiroyuki Kayata, Koji Amano, Hideaki Yakushiji, Nobutaka Mukai, Yasuki Nakata, Junichiro Yokota

**Affiliations:** a Sakai City Medical Center, Critical Care Medical Center, Department of Acute Care Surgery, 1-1-1 Ebaraji-cho, Nishi-ku, Sakai City, Osaka, 593-8304, Japan; b Department of Orthopedic Surgery, 1-1-1 Ebaraji-cho, Nishi-ku, Sakai City, Osaka, 593-8304, Japan

**Keywords:** CT, computer tomography, IPTR, iliopubic tract repair, mIPOM, modified intraperitoneal onlay mesh technique, OTA, Orthopedic Trauma Association, TAPP, transabdominal preperitoneal, TAWH, traumatic abdominal wall hernia, TEP, total extraperitoneal, Iliopubic tract repair, Inguinal hernia, Laparoscopic surgery, Pelvic fracture, Modified intraperitoneal onlay mesh

## Abstract

**Introduction:**

Pelvic fractures can occur in minor injuries, such as falls, in the elderly. Extensive adhesion of preperitoneal space is common after pelvic fracture surgery; hence, surgical interventions for inguinal hernia may be challenging. We treated a case of inguinal hernia after pelvic fracture surgery, using novel laparoscopic methods: iliopubic tract repair (IPTR) and modified intraperitoneal onlay mesh (mIPOM) approach.

**Presentation of case:**

This is the case of an elderly male with pelvic fracture. Open reduction and internal fixation were performed. Eighteen months after the procedure, a right inguinal bulge appeared, swelling increased, and he opted for surgery. We chose laparoscopic surgery to determine the status of the hernia and anatomy around the pelvis. He was diagnosed with an indirect inguinal hernia, and the inner inguinal ring was widely open. We chose the mIPOM approach and IPTR. He was discharged on day 3 post-operation. He developed a seroma after surgery, which disappeared after a month. Six months post-operation, no recurrence or neurologic pain observed.

**Discussion:**

The transabdominal preperitoneal approach (TAPP) was initiated at first; however, the adhesion inside the inferior epigastric vessels was very strong, challenging to break into the preperitoneal space. We switched to the mIPOM method because the peritoneum was fragile and difficult to suture. Additionally, the internal ring was widely opened; hence, we proceeded with IPTR on confirmation that no tension on the abdominal wall was applied.

**Conclusions:**

Laparoscopic surgery is useful in flexibility of surgical options, such as TAPP, IPTR, IPOM, in addition to hybrid conversion.

## Highlights

• Surgery in elderly patients with pelvic fracture are expected to rise in the future.
• Surgery for inguinal hernia can be challenging due to extensive adhesion formation.
• Effective novel laparoscopic methods used: laparoscopic IPTR and mIPOM approach.
• Laparoscopic surgery useful in flexibility of surgical options (TAPP, IPTR, IPOM).

## 1 Introduction

Pelvic fractures can occur both in high-intensity traumas, such as traffic accidents and crashes, and minor injuries, such as falls, in the elderly with bone vulnerability. Hence, in this population, dedicated surgical procedures are expected to rise in the future. The incidence of inguinal hernia is a rare complication after pelvic fracture surgery [1].

In addition to hematoma formation and tissue damage due to the fracture, pelvic fracture surgery involves preperitoneal space dissection and surgical field expansion. Additionally, advanced adhesion is common after the surgery. Thus, surgical procedures for inguinal hernia after pelvic fracture surgery may be challenging.

The indication for laparoscopic surgery in patients with a history of lower abdominal surgery or recurrent hernia remains controversial [2].

We treated a case of inguinal hernia that developed after pelvic fracture surgery using a laparoscopic iliopubic tract repair (IPTR) and modified intraperitoneal onlay mesh (mIPOM) approach. This case report has been reported in line with the SCARE criteria [3].

## 2 Presentation of case

An 81-year-old man was admitted to our hospital due to a fall from a 1-m step. He has no medical history and a stable hemodynamic profile. Abdominal computed tomography (CT) showed a pelvic (acetabulum) fracture that corresponds to the

**Fig. 1:**
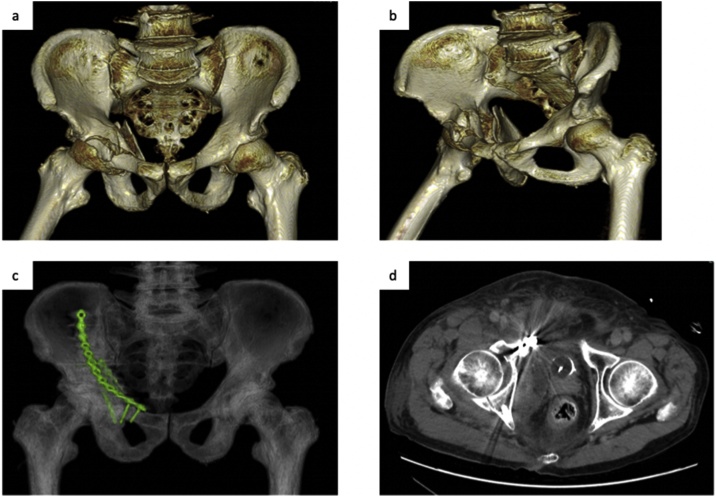
Pre- (a, b) and post-operative (c, d) computed tomography. a, b: Three-dimensional reconstructive computed tomography (CT) showing the right pelvic (acetabulum) fracture. Both anterior and posterior column are fractured. c: Open reduction and internal fixation (ORIF) was performed. d: CT showed retroperitoneal hematoma and no evidence of inguinal hernia.

**Fig. 2:**
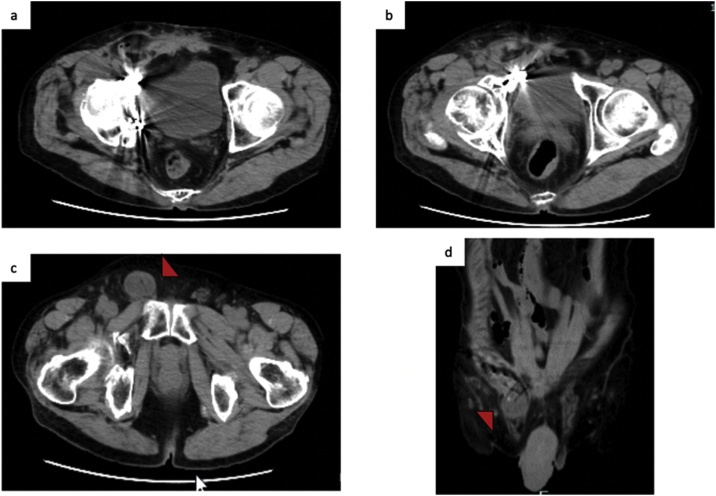
Computed tomography 18 months after pelvic fracture surgery. a, b: The identification of hernia orifice is difficult due to plate artifacts. c, d: The hernia sac reaches the scrotum and small bowel was incarcerated (red triangle).

**Fig. 3:**
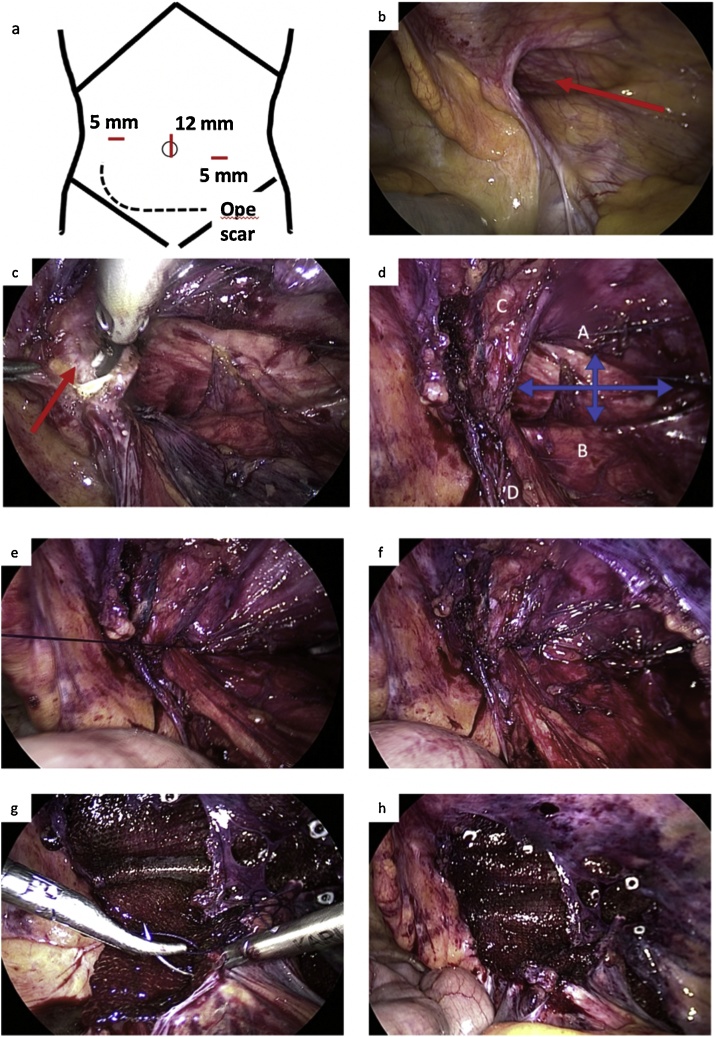
Operative findings. a: Trocar placement. b: Hernia orifice (red arrow). Type I-2 (Japan Hernia Society) or type PL2 (European Hernia Society). c: Strong adhesion of retroperitoneum around pubis (red arrow). d: Completion of the dissection and widely extended internal ring (blue arrow): A, transversalis fascial arch; B, iliopubic tract; C, inferior epigastric vessels; and D, vas deferens. e, f: Iliopubic tract repair. Approximation of the transversalis arch with iliopubic tract with interrupted sutures: the first stitch (e) and the completion (f). g, h: Modified intraperitoneal onlay mesh: the mesh was fixed by tacker and was running sutured at the inferior-medial corner (g). Completion of mesh fixation (h).

AO/Orthopedic Trauma Association classification type 62-C1 (Fig. 1a, b), with extravasation of contrast medium [4]. Emergency angiography was performed to embolize the right obturator artery, internal pudendal artery, and lower gluteal artery. Pelvic fracture surgery was performed 4 days after injury. Open reduction and internal fixation were performed using the ilioinguinal approach and modified Stoppa approach [5] (Fig. 1c, d). The postoperative course was effective with no complications. Eight months after the operation, a right inguinal bulge appeared. He was diagnosed with an inguinal hernia and, therefore, recommended for surgery. However, he decided to follow the progression of the hernia; eventually, the swelling and feeling of discomfort increased and opted to undergo a surgery 18 months after the initial pelvic surgery. The hernia was incarcerated several times, but finger reduction of the hernia was easily achieved. Preoperative CT showed that the hernia reached the scrotum and enlarged the density of soft tissue around the pubis (Fig. 2). Laparoscopic surgery was chosen to confirm the status of the hernia and anatomy around the pelvis from the abdominal cavity. The operation was performed under general anesthesia and pneumoperitoneum with the trocar arrangement placed as shown in Fig. 3a. There was no intra-abdominal adhesion. The patient was diagnosed with an indirect inguinal hernia that corresponds to the European Hernia Society classification type PL2 or Japan Hernia Society classification type I-2, and the inner inguinal ring was widely opened (Fig. 3b). The transabdominal preperitoneal (TAPP) approach was initiated; however, the adhesion inside the inferior epigastric vessels was very strong, making it challenging to break into the preperitoneal (Retzius) space (Fig. 3c). We switched to the mIPOM method because the peritoneum was fragile and difficult to suture. In addition, the internal ring was widely opened; hence, we proceeded with IPTR on confirmation that no tension on the abdominal wall was applied. The repair was achieved by approximating the transversalis arch along the iliopubic tract with 4 interrupted sutures using 3-0 Vicryl (Ethicon, Somerville, NJ) (Fig. 3e, f). Ventralight ST mesh (10.2 × 16.2 cm) (Davol Inc, Subsidiary of C. R. Bard, Inc. Warwick, RI) was placed to cover the sutured internal hernia ring, especially on the lateral side, and was secured to the transverse abdominal muscle using the CapSure Fixation System (Davol Inc, Subsidiary of C. R. Bard, Inc. Warwick, RI). On the dorsal side, the mesh was covered with the peritoneum and fixed with a 3-0 Vicryl continuous suture (Fig. 3g, h). The 12-mm trocar site was closed with 2-0 Vicryl and the skin was closed with 4-0 PDS (Ethicon Inc; Johnson & Johnson, Somerville, NJ). The operating time was 135 min and bleeding were limited. He was discharged on day 3 after surgery. He developed a seroma after the surgery, but disappeared approximately a month later. Six months after the operation, there was no recurrence or neurologic pain.

## 3 Discussion

We experienced a rare case of inguinal hernia after pelvic fracture surgery that was treated using a laparoscopic approach.

A traumatic abdominal wall hernia (TAWH) due to direct external force has been reported as an inguinal hernia associated with a pelvic fracture [6,7]. However, in this case, there was no muscle defect or signs of hernia observed at the time of the pelvic fracture surgery. Furthermore, the patient was diagnosed with an external inguinal hernia. Therefore, the hernia, in this case, was not a TAWH. During the pelvic fracture surgery, the surgical field was developed using both the ilioinguinal approach and modified Stoppa approach [5]. The inguinal ligament, conjoint tendon, iliopubic tract, and rectus abdominis muscle attachment were incised, and the Retzius space was widely deployed in the surgical field. It was presumed that the hernia developed due to the destruction of the inguinal canal and musculoskeletal vulnerability of the elderly. We initiated the surgery in anticipation of the onset of direct or supravesical hernia due to cutting of the rectus abdominal muscle attachment site. However, there were no signs of either direct or supravesical hernia due to strong adhesion around the pubis.

In this case, it is like a recurrent inguinal hernia occurred after both anterior and posterior approach repair due to a history of pelvic fracture surgery and opened inguinal canal. The latest guideline recommended that “in patients with pelvic scarring due to radiation or pelvic surgery, consider an anterior approach.” and “the choice of technique depends on patient- and surgeon-specific factors” [2]. We had two reasons for the choice of laparoscopic surgery. First, as this was a case of atypical inguinal hernia, we wanted to confirm the state of the hernia and anatomy around the pelvis from the abdominal cavity. Second, we had three options available, including TAPP, IPOM, and an open conversion (Hybrid approach), by performing laparoscopic surgery. We had treated a surgical case of inguinal hernia that developed after pelvic fracture surgery, using the same ilioinguinal and modified Stoppa approach, and the Retzius space was easily detached and TAPP was completed. From this standpoint, because each situation is unique, the availability of multiple options for laparoscopic surgery is considered useful.

IPOM is not recommended for primary inguinal hernia because of its high recurrence rate [8]; however, its usefulness as an alternative method when TAPP and TEP cannot be performed has been reported [9,10]. IPTR has been reported to be useful when a mesh cannot be used in a contamination procedure or when an internal ring is enlarged in children [11,12]. For this procedure, we chose mIPOM for two reasons. First, the peritoneum was fragile due to postoperative adhesions and could not be sutured. Second, it was difficult to break into the Retzius space inside the inferior epigastric vessels. To avoid the risk of bulging of the mesh, we proceeded with IPTR after confirming that the abdominal wall was not tensioned. Furthermore, in order to prevent recurrence due to turning up of the mesh on the dorsal side, the mesh was placed under the peritoneum and sutured and fixed. As a result, we considered that the procedure was like the IPOM-Plus method used for abdominal incisional hernia [13].

With an aging society, pelvic fracture cases due to minor injuries in the elderly are expected to rise in the future [14]. Thus, the number of inguinal hernia cases subsequent to pelvic fractures may increase.

This was our first case and the long-term prognosis was, therefore, not determined. It would be necessary to accumulate additional cases. Each case is unique; hence, it is necessary to respond to patient’s needs flexibly during the surgical procedures. For this reason, a laparoscopic approach would be useful.

## 4 Conclusion

Laparoscopic surgery is useful in the diagnosis of hernia, confirmation of the anatomy from the abdominal cavity, and flexibility of surgical options, such as TAPP, IPTR, IPOM, in addition to hybrid conversion.

## Sources of funding

This work did not receive any specific grant from funding agencies in the public, commercial, or not-for-profit sector.

## Ethical approval

This is a case report and it didn’t require ethical approval from ethics committee according to our institution.

## Consent

The patient provided permission for us to publish features of the case, and the patient’s identity has been protected. Written informed consent was obtained from the patients for publication of these case report.

## Author contribution

Yusuke Tsunetoshi takes full responsibility for the work represented in this manuscript (conception of study, acquisition, analysis and interpretation of data) and drafted the manuscript.

Yusuke Tsunetoshi, Koichi Inukai, Masanori Kawamoto, and Koji Amano contributed to performing the surgery.

Akihiro Usui critically revised the manuscript.

All authors read and approved the final manuscript.

## Registration of research studies

This is a case report study.

## Guarantor

Yusuke Tsunetoshi.

## Provenance and peer review

Not commissioned, externally peer-reviewed.

## Declaration of Competing Interest

All authors have no conflicts of interests.
